# The association between triglyceride glucose-waist-to-height ratio and non-alcoholic fatty liver disease in adults aged over 60 in the United States: a cross-sectional study

**DOI:** 10.3389/fpubh.2025.1569324

**Published:** 2026-01-21

**Authors:** Lixian Lin, Guoyan Pan, Lijun Liu, Junwei Huang

**Affiliations:** 1 Department of Gastroenterology, The First Hospital of Putian City, Putian, Fujian, China; 2 Department of Cardiology, The First Hospital of Putian City, Putian, Fujian, China

**Keywords:** NAFLD, NHANES, obesity indicator, TyG, TyGWHtR

## Abstract

**Objectives:**

To identify the optimal surrogate indicator for the initial public health screening of non-alcoholic fatty liver disease (NAFLD) among U.S. adults aged 60 years and above, by systematically comparing the predictive performance of a novel index—the triglyceride glucose-waist-to-height ratio (TyGWHtR)—against 15 other conventional obesity, metabolic, and inflammatory indices.

**Method:**

This cross-sectional study included 1,634 participants from the National Health and Nutrition Examination Survey (2017-March 2020). This study included 16 indicators: weight, waist-to-height ratio (WHtR), body mass index (BMI), waist circumference (WC), weight-adjusted-waist index (WWI), triglyceride glucose (TyG), TyGBMI, TyGWHtR, TyGWWI, TyGWC, TyGWeight, cardiometabolic index (CMI), systemic inflammatory response index (SIRI), systemic immune inflammation index (SII), visceral adiposity index (VAI), and lipid accumulation product (LAP). The participants were categorized into a non-NAFLD group (*n* = 945) and an NAFLD group (*n* = 689). The associations between 16 potential indicators and NAFLD, as defined by FibroScan®, were evaluated using weighted multivariable logistic regression. Indicators that displayed significant associations were further analyzed using receiver operating characteristic (ROC) curves. To investigate a dose-response relationship, we employed restricted cubic splines. Additionally, subgroup and sensitivity analyses were conducted to assess the robustness of the findings.

**Result:**

Five indicators (WHtR, TyG, TyGWHtR, WWI, and CMI) showed significant positive associations with NAFLD. Among these, TyGWHtR demonstrated the best predictive performance [area under the curve (AUC): 0.7641; 95% CI: 0.7414–0.7869], with a nonlinear dose-response relationship. The association remained robust across all subgroups and sensitivity analyses.

**Conclusion:**

The TyGWHtR index is a simple, low-cost, and effective single indicator that outperforms multiple conventional indices for identifying older U.S. adults at high risk of NAFLD. Its reliance on routinely measured clinical parameters makes it an efficient tool for initial risk stratification in community and public health settings, facilitating targeted prevention and supporting healthy aging initiatives.

## Introduction

The global population is aging at an unprecedented rate, primarily due to advancements in medicine and public health. While this represents a significant societal achievement, it also poses profound challenges for healthcare systems worldwide. The number of older adults aged 60 and above is rapidly increasing and is projected to exceed 2.1 billion by 2050 (1). Many individuals over 60 often develop multiple comorbidities as they age, including cardiovascular diseases, NAFLD, and chronic obstructive pulmonary disease (2). The combination of aging and these comorbidities creates complex challenges for public health, healthcare systems, and economies, particularly as treatment and care management become more complicated, leading to higher health costs and increased demand for long-term care (3, 4). Among the various age-related health challenges, liver diseases, especially NAFLD, have emerged as a significant public health concern (5).

NAFLD, also known as metabolic dysfunction-associated fatty liver disease, comprises a group of chronic progressive conditions caused by overnutrition and insulin resistance (IR), contributing to a public health crisis. It includes non-alcoholic fatty liver, non-alcoholic steatohepatitis, as well as related liver fibrosis and cirrhosis (6). With the increase in global obesity rate and the incidence of type 2 diabetes, the prevalence of NAFLD is also increasing yearly (7, 8). Epidemiological studies indicate that the global incidence rate of NAFLD is as high as 37.8%, with males (39.7%) having a higher rate than females (25.6%) (9). Systematic research shows that Latin America ranks first with an incidence rate of 44.4% (10).

NAFLD is associated with various clinical diseases and poses significant risks. Research indicates that NAFLD and diabetes have a mutual causal relationship, and NAFLD is also closely linked to cardiovascular disease, liver dysfunction, chronic kidney disease, and an increased risk of cancer (11–14). Over time, NAFLD has become the leading risk factor for liver cirrhosis and liver cancer, surpassing hepatitis B virus infection (15). In the United States, a significant amount of money is spent each year on the diagnosis and treatment of NAFLD and its associated complications, so early management and intervention of people at high risk of NAFLD is a very important public health issue (16). Given the expanding population of older adults, the complexity of managing age-related multimorbidity, and the growing need for cost-effective public health strategies, identifying a simple predictive tool for early detection of high-risk individuals is crucial. This is essential to alleviate the clinical, public health, and economic burdens of NAFLD and to promote healthy aging.

NAFLD is characterized by the accumulation of fat in more than 5% of liver cells, excluding cases of alcohol abuse (defined as over 30 grams per day for males and over 20 grams per day for females) and any clear history of liver disease (17). In NAFLD patients, the presence of IR in the body allows insulin to stimulate excessive *de novo* lipogenesis and very low-density lipoprotein production in liver cells, leading to increased triglyceride (TG) synthesis. Additionally, during inflammation, the impaired TG excretion pathway further accumulates TG within the liver. The reduced oxidation, utilization, and transport of TG exacerbate hepatic steatosis, ultimately resulting in hepatocyte damage, fibrosis activation, and lobular inflammation necrotizing (18–20).

Traditionally, the gold standard for diagnosing NAFLD has been liver biopsy. However, this method is expensive and invasive, carries risks for patients, and contravenes the Helsinki Principles, making it unsuitable for large-scale health screenings in the general population (21). With advancements in medical science, abdominal ultrasound has emerged as a cost-effective, safe, and commonly used method for screening fatty liver, as recommended by the Asia Pacific guidelines (22). However, due to the complexity of the ultrasound diagnostic criteria for NAFLD (23), its widespread application in public screening still faces limitations in terms of complexity and economic cost (24, 25). Therefore, there is significant public health value in exploring easy-to-use, practical, and reliable indicators of NAFLD. This pursuit has focused on two main categories of readily available data: anthropometric measures and biochemical/hematological indices.

In public health practice, various indicators are used to assess obesity. Traditional measurements include weight, waist circumference (WC), waist-to-height ratio (WHtR), and body mass index (BMI). Additionally, newer indicators such as the weight-adjusted waist index (WWI), visceral adiposity index (VAI), and lipid accumulation product (LAP) are being explored. Many of these indicators have been shown to correlate with the risk of NAFLD (26).

In addition, inflammation plays a crucial role in the development of NAFLD. Under normal conditions, the gut microbiota influences lipid metabolism by regulating the absorption and processing of dietary fats and cholesterol. When inflammation occurs, it can lead to an imbalance in the gut microbiota. Endotoxins trigger an inflammatory cascade through the gut-liver axis, leading to the production of reactive oxygen species, cell apoptosis, and immune dysregulation. This, in turn, exacerbates the body's inflammatory state and lipid metabolism abnormalities, ultimately contributing to the onset of NAFLD (27–29). We can also try to predict the risk of NAFLD onset by looking at inflammatory markers. Currently, two new inflammatory markers, systemic inflammatory response index (SIRI) and systemic immune inflammation index (SII), have been proposed, with SII being associated with hepatic steatosis but not with liver fibrosis (30, 31). However, it remains uncertain whether SIRI or SII is associated with NAFLD in older population.

Beyond anthropometric measures, indices reflecting underlying metabolic dysfunction, such as IR, are crucial for understanding NAFLD pathogenesis. The triglyceride glucose index (TyG) and its related indicators, such as the triglyceride glucose-waist circumference (TyGWC) and the triglyceride glucose-waist-to-height ratio (TyGWHtR), are emerging markers that reflect IR. An increase in these indices often suggests the presence of metabolic abnormalities (32). Due to their accessibility and ease of calculation, TyG-related indices have shown considerable utility in clinical and public health contexts. For example, TyG is associated with cardiovascular mortality risk in patients with coronary heart disease and hypertension (33). Additionally, the TyGWC is linked to a higher risk of myocardial infarction (34), while the TyGWHtR can predict hypertension risk in American adults (35). A study conducted on the general population in Japan found that the triglyceride glucose-body mass index (TyGBMI) is an effective predictor of NAFLD (36). However, there is currently a lack of comprehensive evaluations regarding NAFLD risk prediction indicators specifically for the population in the United States aged 60 and older. This gap highlights the need to identify a simple, yet highly effective indicator tailored for this growing demographic.

Applying all the many existing NAFLD-associated indicators in population screening is often impractical due to cost and complexity—a common challenge in public health. Consistent with the principle proposed by Andrus et al. that simple models using routine data are preferable for primary care screening (37), this study aimed to determine whether the novel TyGWHtR index serves as a superior single indicator for NAFLD risk stratification among U.S. adults aged ≥60 years, by systematically evaluating its association with NAFLD and comparing its performance against 15 other common clinical indices.

## Method

### Study populations

The National Health and Nutrition Examination Survey (NHANES) is a cross-sectional study aimed at assessing the health and nutritional status of American residents. This initiative is overseen by the National Center for Health Statistics (NCHS) and is part of the Centers for Disease Control and Prevention (CDC). The survey is conducted biennially and uses stratified sampling to gather data that accurately represents the U.S. population. Since 2017, NHANES has employed ultrasound methods to collect liver data, so our study includes data from the cycle of 2017 to March 2020. The NHANES survey protocol was approved by the NCHS Institutional Ethics Review Board, and all participants signed written informed consent. The data for this study were obtained entirely from the publicly accessible NHANES database. All datasets have been anonymized and do not include any personal identifying information. This study adheres strictly to the NHANES data use protocol and does not incorporate any restricted or sensitive data. All data used in this study are publicly available on the official NHANES website.^1^

**Figure 1:**
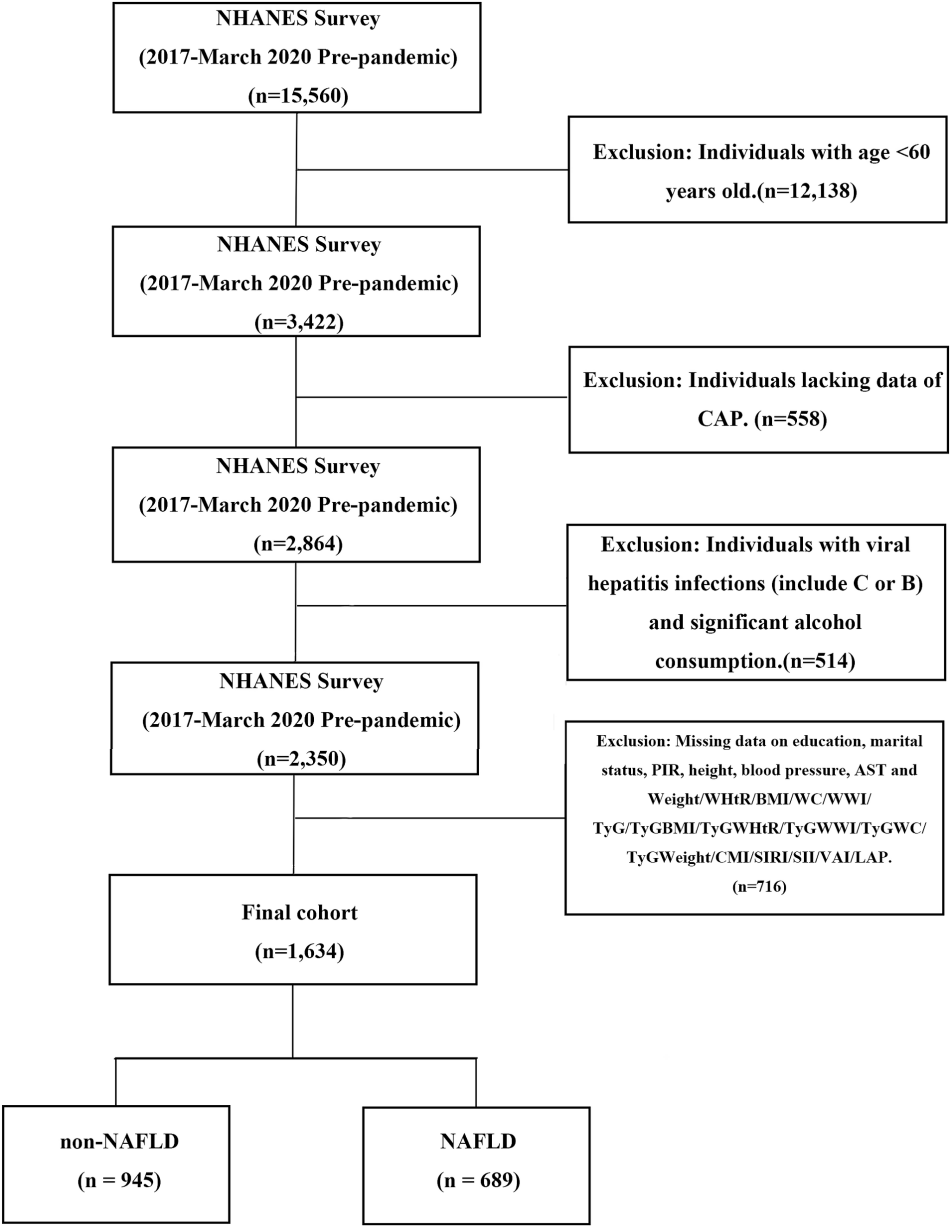
Displays a detailed flow chart that the screening process utilized to select eligible participants in NHANES survey (2017 - March.2020 Pre-pandemic). NHANES, National Health and Nutrition Examination Survey; CAP, controlled attended parameter; BMI, body mass index; WC, waist circumference; WHtR, waist-to-height ratio; CMI, cardiometabolic index; WWI, weight-adjusted-waist index; TyG, triglyceride-glucose index; TyGWWI, triglyceride glucose-weight adjusted waist index; TyGBMI, triglyceride glucose-body mass index; TyGWHtR, triglyceride glucose-waist-to-height ratio. TyGWC, triglyceride glucose-waist circumference; TyGWeight, triglyceride glucose-weight; SIRI, systemic inflammatory response index; SII, systemic immune inflammation index; LAP, lipid accumulation product; VAI, visceral adiposity index; NAFLD, nonalcoholic fatty liver disease.

The survey initially included 15,560 individuals. Participants under the age of 60 were excluded (*n*=12,138). Subsequently, those lacking controlled attended parameter (CAP) data were also excluded (*n*=558). Following the NAFLD diagnostic criteria, we excluded participants with hepatitis (including those who tested positive for hepatitis B antigen, hepatitis C RNA, or hepatitis C antibody) and those who reported significant alcohol consumption (defined as consuming four or more drinks per day) (*n*=514). Subsequently, data that lacked information on education, marital status, body measurements, blood pressure, aspartate aminotransferase (AST), and that could not be used to calculate the obesity index were excluded (*n*=716). Ultimately, 1,634 participants were included in the study. Participants were divided into a non-NAFLD group (*n*=945) and an NAFLD (*n*=689) group. The detailed flow chart of participant selection is illustrated in Figure 1.

### Independent variable

The independent variables of this study include weight, WHtR, BMI, WC, WWI, TyG, TyGBMI, TyGWHtR, TyGWWI, TyGWC, TyGWeight, CMI, SIRI, SII, VAI, and LAP. The data for WC, BMI, and weight were obtained directly from the database. The remaining variables were calculated using specific formulas. (Note: To convert triglycerides, 1 mmol/L is equal to 0.0113 mg/dL. Abbreviation: HDL-C stands for high-density lipoprotein cholesterol).

$$\text{WHtR}=\frac{\mathrm{WC}\;(\mathrm{cm})}{\text{Height}\;(\mathrm{cm})}$$

$$\mathrm{WWI}=\frac{\mathrm{WC}(\mathrm{cm})}{\sqrt{\text{weight}}(\mathrm{kg})}$$

TyG=ln[TG(mg/dL)×Glucose(mg/dL)/2]

TyGBMI=TyG×BMI

TyGWC=TyG×WC

TyGWWI=TyG×WWI

TyGWeight=TyG×Weight

TyGWHtR=TyG×WHtR

CMI=TG(mmol/L)/HDL−C(mmol/L)×WHtR

SIRI=monocyte count×neutrophils count/lymphocytes count

SII=platelet count×neutrophil count/lymphocyte count

VAI=WC (cm)/(39.68 + 1.88 x BMI (kg/m2)) x TG (mmol/L)/1.03 x 1.31/HDL-C (mmol/L) for males.

VAI=WC (cm)/(36.58 + 1.89 x BMI (kg/m2)) x TG (mmol/L)/0.81 x 1.52/HDL-C (mmol/L) for females.

LAP=[WC(cm)−65]xTG(mmol/L)for males

LAP=[WC(cm)−58]xTG(mmol/L)for females

### Dependent variable

The dependent variable in this study is the presence of NAFLD. In the NHANES dataset, participants' livers were examined using the FibroScan® model 502 V2 Touch, which was equipped with either a medium (M) or extra-large (XL) hand probe in the Mobile Examination Center. This device can simultaneously measure ultrasound attenuation related to hepatic steatosis and record the CAP^TM^, which serves as an indicator of liver fat content. According to previous studies, NAFLD is defined as a CAP of ≥285 dB/m, which provides 80% sensitivity and 77% specificity (38, 39).

### Covariates of interest

Based on the etiology of NAFLD and clinical comorbidities, covariate data were included in this study, encompassing demographic data (gender, age, marital status, education, and race), poverty-to-income ratio (PIR), smoking status, coronary heart disease (CHD), chronic heart failure (CHF), hypertension, diabetes, AST, and creatinine. All data were collected and measured using standardized methods through interviews with professionally trained and certified staff.

Marital status was categorized as “married/Living with Partner” or “other (including widowed, divorced, separated, never married)”. Education levels were classified as follows: less than high school, high school (which includes 9-11th grade, along with 12th grade without a diploma, as well as high school graduates/GED or equivalent), and higher than high school (which includes some college or AA degree, College graduate or above). Race was categorized as Mexican American, Non-Hispanic Asian, Non-Hispanic Black, Non-Hispanic White, Other Hispanic, and Other Race - Including Multi-Racial. Participants were considered to have a history of smoking if they affirmed the following question: “Smoked at least 100 cigarettes in life?” Additionally, complications, including CHF and CHD, were self-reported by participants. According to the questionnaire of participants, diabetes was classified into “yes/borderline/no”. Hypertension was diagnosed if the participant had an average SBP of 140mmHg and/or DBP of 90mmHg. Additionally, if participants self-reported a history of hypertension or the use of hypertension medication in the section “Questionnaire”, they were also classified as hypertensive.

### Statistical analysis

All statistical analyses were performed by NHANES analytic guidelines (40). Continuous variables were reported as median (Interquartile range, IQR). The number of cases and the composition ratio [*n* (%)] were used to describe the categorical data. “*n*” was considered as an unweighted number, and “(%)” was considered as a weighted percentage. Considering the complex survey design, we employed a weighted linear regression model to analyze the differences in continuous variables between the groups, conducting a Wald test on the regression coefficients. The comparison of categorical variables is conducted using the weighted chi-square test. The association between indicators and the risk of NAFLD was assessed using weighted multivariate logistic regression analysis, with results presented as odds ratios (OR) and 95% confidence intervals (CI). To account for potential covariates, three models were established for sensitivity analysis: Model 1 was a crude model; Model 2 was adjusted for gender, age, race, education, and marital status; Model 3 was further adjusted for gender, age, race, education, marital status, PIR, smoking, CHF, CHD, hypertension, diabetes, AST, creatinine. Indicators that were not significantly associated with NAFLD (*p* > 0.05) or had poor association (OR = 1) were excluded from the subsequent analysis. In addition, Bonferroni correction was applied to *P* values to reduce the Type I error.

We plotted the receiver operating characteristic (ROC) curves to evaluate the effectiveness of five indicators in enhancing the detection of NAFLD. We reported the area under the curve (AUC), cut-off values, specificity, and sensitivity for each indicator. We selected TyGWHtR, which had the largest AUC value, as the reference, and then used the DeLong test to compare it with other indicators to confirm its optimal predictive performance before proceeding with the follow-up analysis. In order to verify the results, we also conducted a 10-fold cross-validation analysis on all five indicators.

The nonlinear analysis between indicators and NAFLD was depicted using the restricted cubic spline (RCS) model. According to the common practice of international scholars (41, 42), the models were conducted with four knots at the 5th, 35th, 65th, and 95th percentiles of the indicator. The covariates adjustment of the RCS model was the same as Model 3.

Subgroup analysis was performed for sensitivity analysis by stratifying the data according to gender (male, female), age (60-69, 70-80), race, education, marital status, smoking, CHF, CHD, hypertension, and diabetes. The differences among the subgroups were illustrated by the *P*-values for interactions.

All analyses were conducted using R version 4.4.1 (released on June 14, 2024), and a two-sided *P* value of <0.05 was considered statistically significant.

## Results

### Baseline characteristics

The baseline characteristics of the study population are summarized in Table 1. The weighted median age of the participants was 68 years, with 760 males accounting for 41.4%. The weighted overall incidence of NAFLD in the study population was 41.8%. Factors associated with a higher likelihood of developing NAFLD included being younger, male, married or living with a partner, identifying as Mexican American, non-Hispanic White, or other races (including multi-racial individuals), as well as having greater values in height, weight, BMI, WC, WHtR, CMI, WWI, VAI, LAP, TyG, and other related indices such as TyGWWI, TyGBMI, TyGWHtR, TyGWC, TyGWeight (*P* <0.05). The incidence of diabetes and hypertension was higher in the NAFLD group. In terms of laboratory results, the NAFLD group exhibited lower levels of HDL-C while showing elevated levels of AST, glucose, TG, monocytes, and neutrophils compared to the non-NAFLD group (P < 0.05).

**Table 1:** Demographic characteristics of the weighted baseline

Variables	Total (*n* = 1,634)	non-NAFLD (*n* = 945)	NAFLD (*n* = 689)	*p*
	*N* = 25,328,849	*N* = 14,732,495	*N*= 10,596,354	
Age, year	68.0 (63.0, 75.0)	69.0 (63.0, 77.0)	67.0 (63.0, 74.0)	**0.029**
Male, *n* (%)	760 (41.4)	416 (37.3)	344 (47.1)	**0.007**
Education, *n* (%)				0.878
High school	558 (33.8)	326 (33.4)	232 (34.3)	
Higher than high school	937 (62.6)	537 (62.8)	400 (62.3)	
Less than high school	139 (3.6)	82 (3.8)	57 (3.4)	
Marital status, *n* (%)				**0.001**
Married/Living with Partner	945 (64.4)	509 (58.9)	436 (72.0)	
Other state	689 (35.6)	436 (41.1)	253 (28.0)	
Race, *n* (%)				**0.006**
Mexican American	133 (3.3)	57 (2.4)	76 (4.5)	
Non-Hispanic Asian	149 (4.4)	90 (4.7)	59 (4.1)	
Non-Hispanic Black	400 (8.3)	256 (9.5)	144 (6.6)	
Non-Hispanic White	739 (76.4)	423 (76.3)	316 (76.5)	
Other Hispanic	163 (4.9)	93 (5.1)	70 (4.6)	
Other Race - Including Multi-Racial	50 (2.8)	26 (2.1)	24 (3.8)	
Smoking, *n* (%)	700 (41.2)	393 (41.6)	307 (40.6)	0.816
PIR, %	2.5 (1.4, 4.6)	2.5 (1.4, 4.6)	2.4 (1.4, 4.6)	0.437
SBP, mmHg	131.7 (119.7, 145.7)	131.3 (119.7, 146.7)	132.0 (119.3, 145.0)	0.782
DBP, mmHg	72.7 (65.7, 80.3)	72.0 (65.3, 79.3)	73.7 (66.3, 81.3)	**0.006**
HDL-C, mmol/L	1.4 (1.1, 1.7)	1.5 (1.2, 1.8)	1.2 (1.1, 1.5)	**<.001**
AST, U/L	19.0 (16.0, 23.0)	19.0 (16.0, 23.0)	20.0 (16.0, 24.0)	**0.015**
Glucose, mg/dL	97.5 (90.0, 112.0)	95.0 (88.0, 105.0)	103.0 (93.0, 122.0)	**<.001**
Triglycerides, mmol/L	1.4 (1.0, 1.9)	1.2 (0.9, 1.7)	1.6 (1.2, 2.2)	**<.001**
Creatinine, umol/L	80.4 (67.2, 96.4)	80.4 (68.1, 96.4)	80.4 (66.3, 96.4)	0.426
WBC, 1000 cells/uL	6.7 (5.6, 8.1)	6.4 (5.3, 7.9)	7.0 (5.8, 8.4)	0.363
Lymphocyte, 1000 cells/uL	1.9 (1.5, 2.4)	1.8 (1.5, 2.3)	2.1 (1.6, 2.6)	0.598
Monocytes, 1000 cells/uL	0.6 (0.5, 0.7)	0.5 (0.4, 0.7)	0.6 (0.5, 0.7)	**0.006**
Neutrophils, 1000 cells/uL	3.8 (3.0, 5.0)	3.7 (2.9, 4.7)	4.1 (3.2, 5.1)	**0.001**
PLT, 1000 cells/uL	228.0 (191.0, 266.0)	227.0 (190.0, 265.0)	229.0 (193.0, 269.0)	0.358
CAP, dB/m	273.0 (230.0, 316.0)	236.0 (212.0, 262.00)	324.0 (303.0, 352.0)	**<.001**
CHF, *n* (%)	80 (4.90)	37 (3.92)	43 (6.24)	0.114
CHD, *n* (%)	143 (8.75)	75 (7.94)	68 (9.87)	0.381
Diabetes, *n* (%)				**<.001**
Yes	421 (20.8)	176 (13.5)	245 (31.0)	
Borderline	81 (4.2)	38 (2.8)	43 (6.1)	
No	1132 (75.0)	731 (83.7)	401 (62.9)	
Hypertension, *n* (%)	977 (55.8)	517 (49.4)	460 (64.7)	**0.003**
Height, cm	164.4 (157.7, 171.8)	163.5 (156.8, 171.0)	165.0 (158.9, 172.5)	**<.001**
Weight, cm	78.7 (67.4, 91.7)	73.3 (63.0, 84.0)	87.8 (75.7, 101.0)	**<.001**
BMI, kg/m2	28.8 (25.4, 33.2)	27.0 (24.1, 30.4)	31.6 (28.1, 36.1)	**<.001**
WC, cm	102.1 (93.4, 112.3)	97.3 (89.4, 106.0)	110.0 (100.6, 119.1)	**<.001**
WHtR	0.6 (0.6, 0.7)	0.6 (0.5, 0.7)	0.7 (0.6, 0.7)	**<.001**
CMI	0.6 (0.4, 1.0)	0.5 (0.3, 0.8)	0.9 (0.6, 1.3)	**<.001**
WWI	11.5 (11.1, 12.0)	11.4 (10.9, 11.9)	11.7 (11.3, 12.1)	**<.001**
TyG	8.7 (8.4, 9.2)	8.6 (8.3, 8.9)	8.9 (8.6, 9.3)	**<.001**
TyGWWI	100.8 (94.5, 108.0)	98.0 (91.6, 104.6)	105.2 (98.8, 111.7)	**<.001**
TyGBMI	251.8 (220.6, 295.7)	233.7 (204.6, 266.6)	282.8 (247.6, 328.7)	**<.001**
TyGWHtR	5.5 (4.9, 6.1)	5.1 (4.6, 5.7)	5.9 (5.4, 6.6)	**<.001**
TyGWC	892.3 (803.8, 1005.1)	837.4 (756.6, 923.8)	982.1 (883.1, 1086.0)	**<.001**
TyGWeight	691.5 (584.1, 817.5)	628.9 (541.9, 729.0)	786.9 (671.1, 918.6)	**<.001**
SIRI	1.1 (0.7, 1.7)	1.1 (0.7, 1.6)	1.2 (0.7, 1.7)	0.194
SII	445.5 (309.7, 629.9)	450.4 (311.8, 634.5)	443.1 (305.4, 612.0)	0.801
LAP	56.8 (35.8, 88.4)	43.3 (28.8, 67.7)	76.9 (53.0, 109.1)	**<.001**
VAI	1.4 (1.1, 2.8)	1.4 (0.9, 2.3)	2.2 (1.4, 3.3)	**<.001**

“*n*” represents the original sample size, “*N*” represents the weighted sample size. *p* value is the weighted value. The bold values representative *p* value < 0.05. NAFLD, nonalcoholic fatty liver disease; PIR, poverty-to-income ratio; BMI, body mass index; WC, waist circumference; SBP, systolic blood pressure; DBP, diastolic blood pressure; HDL-C, high-Density lipoprotein cholesterol; AST, aspartate aminotransferase; WBC, white blood cell count; PLT, Platelet count; CAP, controlled attenuation parameter; CHF, Chronic heart failure; CHD, Coronary heart disease; WHtR, waist-to-height ratio; CMI, cardiometabolic index; WWI, weight-adjusted-waist index; TyG, triglyceride-glucose index; TyGWWI, triglyceride glucose-weight adjusted waist index; TyGBMI, triglyceride glucose-body mass index; TyGWHtR, triglyceride glucose-waist-to-height ratio. TyGWC, triglyceride glucose-waist circumference; TyGWeight, triglyceride glucose-weight; SIRI, systemic inflammatory response index; SII, systemic immune inflammation index; LAP, lipid accumulation product; VAI, visceral adiposity index.

### Association of indicators with NAFLD

For per-unit increase in TyGWHtR, the risk of developing NAFLD rises (Model 1: OR = 1.29, 95% CI: 1.26 to 1.32). The other four indicators—WHtR, TyG, WWI, and CMI—are also significantly associated with NAFLD (see Table 2). The remaining 11 indicators were excluded from further analysis due to insufficient statistical significance or poor associations (OR approximately equal to 1). All significant associations persisted in both the partially adjusted model (Model 2) and the fully adjusted model (Model 3).

**Table 2:** Multivariable-adjusted odds ratios and 95% confidence intervals for 16 indicators associated with the risk of NAFLD in individuals aged 60 and above.

Indicators	Model 1			Model 2			Model 3		
	OR (95% CI)	*p*	*p’*	OR (95% CI)	*p*	*p’*	OR (95% CI)	*p*	*p’*
Weight	1.01 (1.01 ~ 1.01)	**<0.001**	**<0.001**	1.01 (1.01 ~ 1.01)	**<0.001**	**<0.001**	1.01 (1.01 ~ 1.01)	**<0.001**	**<0.001**
WHtR	11.26 (8.27 ~ 15.31)	**<0.001**	**<0.001**	13.31 (9.71 ~ 18.26)	**<0.001**	**<0.001**	10.72 (6.75 ~ 17.04)	**<0.001**	**<0.001**
BMI	1.04 (1.03 ~ 1.04)	**<0.001**	**<0.001**	1.04 (1.03 ~ 1.04)	**<0.001**	**<0.001**	1.03 (1.03 ~ 1.04)	**<0.001**	**<0.001**
WC	1.02 (1.01 ~ 1.02)	**<0.001**	**<0.001**	1.02(1.01 ~ 1.02)	**<0.001**	**<0.001**	1.02 (1.01 ~ 1.02)	**<0.001**	**<0.001**
WWI	1.19 (1.15 ~ 1.24)	**<0.001**	**<0.001**	1.27 (1.22 ~ 1.32)	**<0.001**	**<0.001**	1.22 (1.17 ~ 1.28)	**<0.001**	**<0.001**
TyG	1.39 (1.32 ~ 1.46)	**<0.001**	**<0.001**	1.38 (1.31 ~ 1.45)	**<0.001**	**<0.001**	1.32 (1.24 ~ 1.41)	**<0.001**	**<0.001**
TyGBMI	1.00 (1.00 ~ 1.00)	**<0.001**	**<0.001**	1.00 (1.00 ~ 1.00)	**<0.001**	**<0.001**	1.00 (1.00 ~ 1.00)	**<0.001**	**<0.001**
TyGWHtR	1.29 (1.26 ~ 1.32)	**<0.001**	**<0.001**	1.30 (1.27 ~ 1.32)	**<0.001**	**<0.001**	1.28 (1.24 ~ 1.33)	**<0.001**	**<0.001**
TyGWWI	1.02 (1.02 ~ 1.02)	**<0.001**	**<0.001**	1.02 (1.02 ~ 1.02)	**<0.001**	**<0.001**	1.02 (1.02 ~ 1.02)	**<0.001**	**<0.001**
TyGWC	1.00 (1.00 ~ 1.00)	**<0.001**	**<0.001**	1.00 (1.00 ~ 1.00)	**<0.001**	**<0.001**	1.00 (1.00 ~ 1.00)	**<0.001**	**<0.001**
TyGWeight	1.00 (1.00 ~ 1.00)	**<0.001**	**<0.001**	1.00 (1.00 ~ 1.00)	**<0.001**	**<0.001**	1.00 (1.00 ~ 1.00)	**<0.001**	**<0.001**
CMI	1.21 (1.16 ~ 1.26)	**<0.001**	**<0.001**	1.20 (1.16 ~ 1.25)	**<0.001**	**<0.001**	1.17 (1.13 ~ 1.21)	**<0.001**	**<0.001**
SIRI	1.03 (0.99 ~ 1.07)	0.190	>0.05	1.02 (0.98 ~ 1.07)	0.323	>0.05	1.00 (0.95 ~ 1.06)	0.898	>0.05
SII	1.00 (1.00 ~ 1.00)	0.799	>0.05	1.00 (1.00 ~ 1.00)	0.892	>0.05	1.00 (1.00 ~ 1.00)	0.979	>0.05
VAI	1.07 (1.05 ~ 1.09)	**<0.001**	**<0.001**	1.07 (1.05 ~ 1.09)	**<0.001**	**<.001**	1.06 (1.04 ~ 1.08)	**<0.001**	**0.002**
LAP	1.00 (1.00 ~ 1.00)	**<0.001**	**<0.001**	1.00 (1.00 ~ 1.00)	**<0.001**	**<.001**	1.00 (1.00 ~ 1.00)	**<0.001**	**0.008**

*p*’ represents the Bonferroni correction P. The bold values representative *p* value < 0.05. Model 1: Crude. Model 2: Gender, Age, Race, Education, Marital status were adjusted. Model 3: Gender, Age, Race, Education, Marital status, PIR, smoking, CHF, CHD, Hypertension, Diabetes, AST, Creatinine were adjusted. OR, Odds Ratio; CI, Confidence Interval; NAFLD, nonalcoholic fatty liver disease; WHtR, waist to height ratio; BMI, body mass index; WC, waist circumference; WWI, weight-adjusted-waist index; TyG, triglyceride-glucose index; CMI, cardiometabolic index; SIRI, systemic inflammatory response index; SII, systemic immune inflammation index; VAI, visceral adiposity index; LAP, lipid accumulation product; PIR, poverty-to-income ratio; CHF, Chronic heart failure; CHD, Coronary heart disease; AST, aspartate aminotransferase.

### Receiver operating characteristic analysis

**Figure 2:**
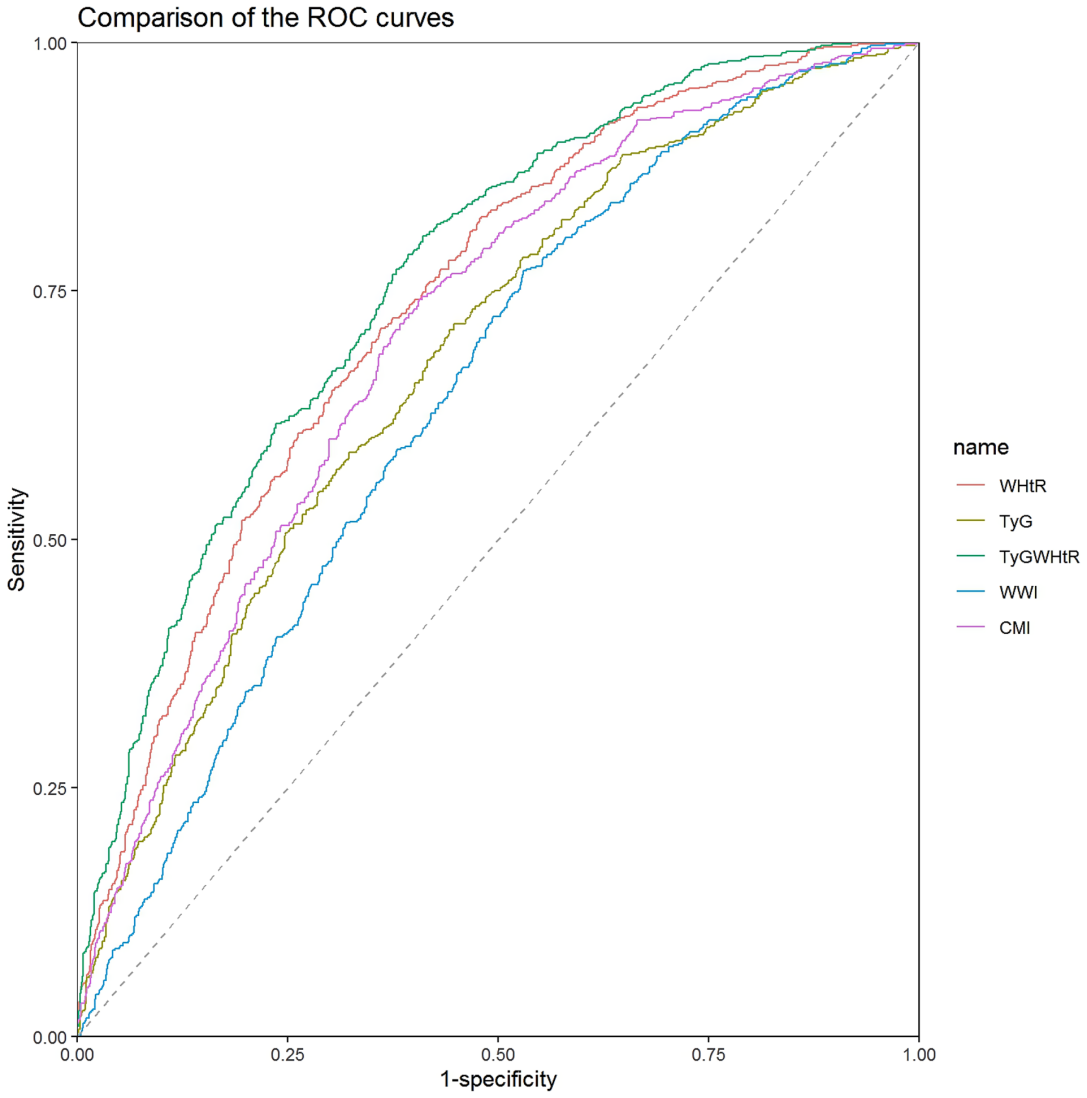
Comparison of the ROC curves of WHtR, TyG, TyGWHtR, WWI, and CMI in total participants. ROC, receiver operating characteristic; WHtR, waist-to-height ratio; TyG, triglyceride-glucose index; TyGWHtR, triglyceride glucose-waist-to-height ratio; WWI, weight-adjusted-waist index; CMI, cardiometabolic index.

The AUC for TyGWHtR in detecting NAFLD was 0.7641 (95% CI: 0.7414-0.7869), which was significantly higher than that of the other four indicators (*P* < 0.001; Table 3 and Figure 2). The results of 10-fold cross-validation indicate that TyGWHtR still has the highest average AUC (0.782), as well as the highest average sensitivity (0.821) and specificity (0.581) (Supplementary Table S1).

**Table 3:** ROC curves for indicators to improve the identification of NAFLD.

Indicators	AUC(95% CI)	*p* value	Cut-off value	Specificity	Sensitivity	*p* for comparison
WHtR	0.7372 (0.7134–0.7611)	**<0.001**	0.620	0.639	0.713	**<0.001**
TyG	0.6810 (0.6552–0.7069)	**<0.001**	8.633	0.552	0.717	**<0.001**
TyGWHtR	0.7641 (0.7414–0.7869)	**<0.001**	5.276	0.589	0.806	Reference
WWI	0.6456 (0.6191-0.6720)	**<0.001**	11.286	0.470	0.771	**<0.001**
CMI	0.7079 (0.6830-0.7329)	**<0.001**	0.572	0.594	0.742	**<0.001**

ROC, Receiver operating characteristic; NAFLD, nonalcoholic fatty liver disease; AUC, Area under the curve; CI, Confidence Interval; WHtR, waist-to-height ratio; TyG, triglyceride-glucose index;TyGWHtR, triglyceride glucose-waist-to-height ratio; WWI, weight-adjusted-waist index; CMI, cardiometabolic index.

### Restricted cubic spline regression analysis

**Figure 3:**
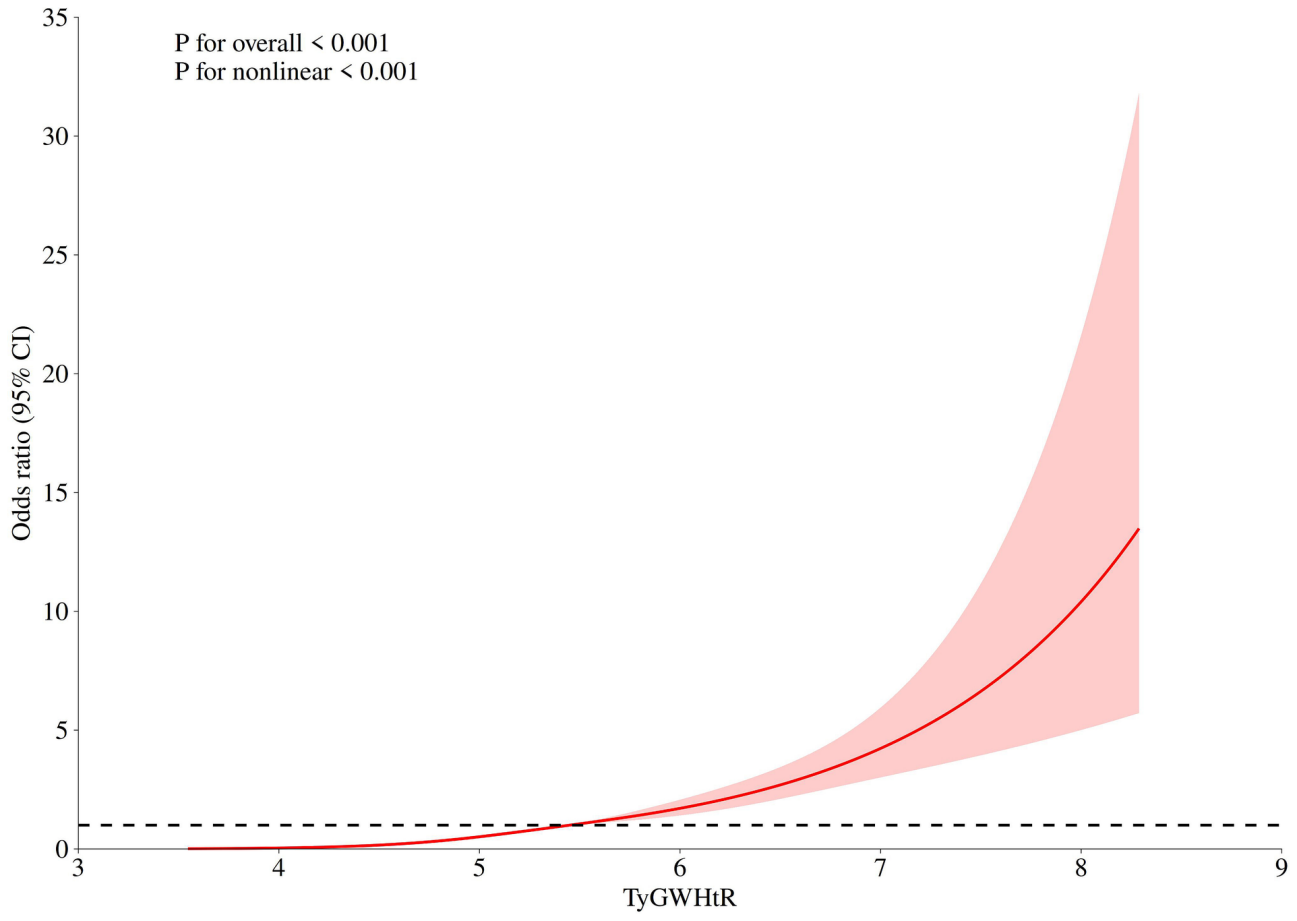
Dose-response relationship between TyGWHtR and the risk of NAFLD. The curve plotted by adjusting for covariates including gender, age, race, education, marital status, PIR, smoking, CHF, CHD, hypertension, diabetes, AST and creatinine. The solid line represents the OR value, and the shadow represents 95% CI. CI, Confidence Interval; RCS, restricted cubic spline; NAFLD, nonalcoholic fatty liver disease; TyGWHtR, triglyceride glucose-waist-to-height ratio; PIR, poverty-to-income ratio; CHF, Chronic heart failure; CHD, Coronary heart disease; AST, aspartate aminotransferase; OR, odds ratio; CI, confidence interval.

After multivariate adjustment, the TyGWHtR showed a positive nonlinear association with NAFLD (*p* for overall <0.001, *p* for nonlinear <0.001, Figure 3). The other four indicators (TyG, WWI, WHtR, CMI) also showed similar nonlinear associations (Supplementary Figure S1).

### Subgroup analysis

**Figure 4:**
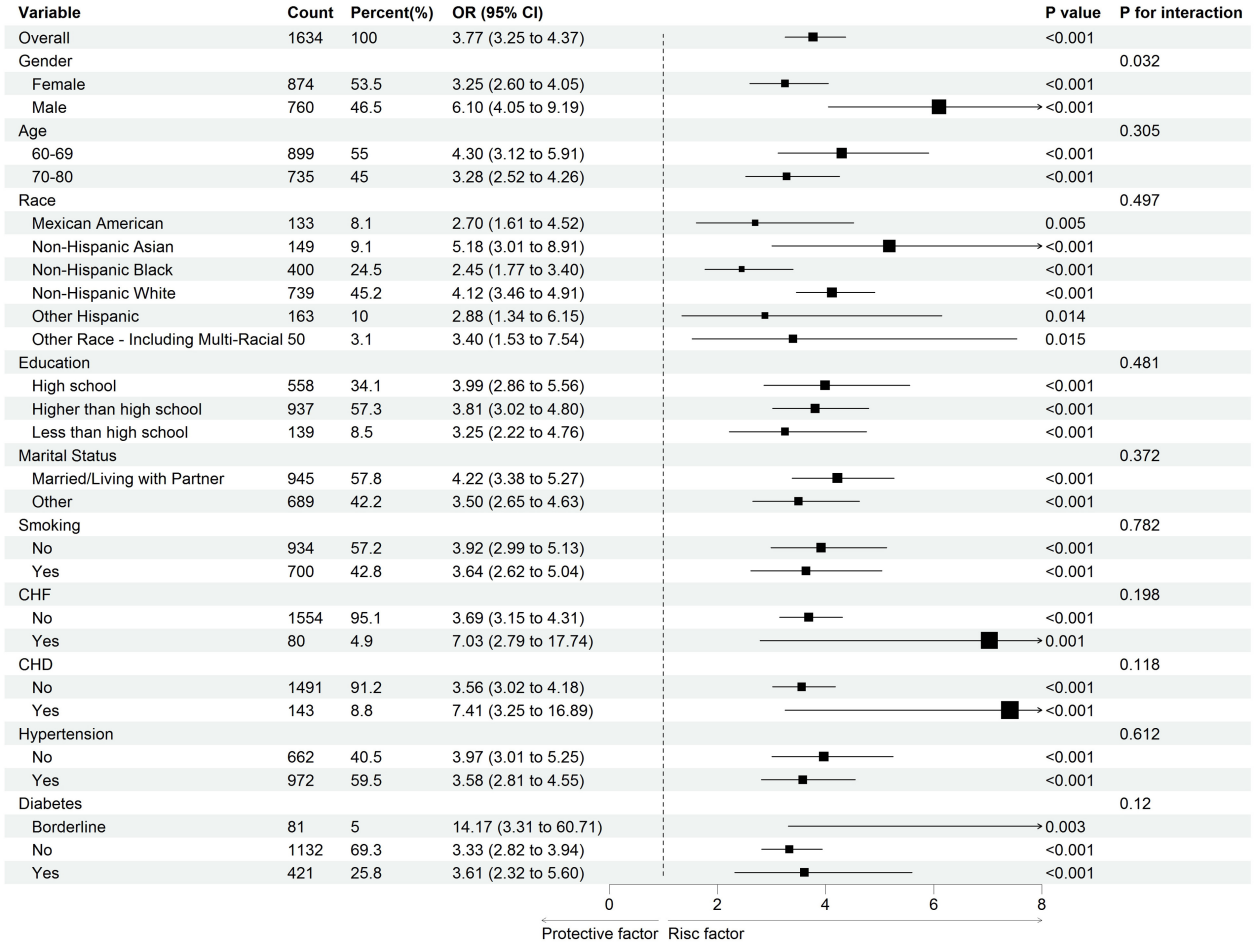
The results of subgroup analysis demonstrate the association between TyGWHtR and the prevalence of NAFLD in US adults aged 60 and above. Weighted logistic regression models were used in both subgroup analysis and forest mapping. OR, odds ratio; CI, confidence interval; CHF, Chronic heart failure; CHD, Coronary heart disease; TyGWHtR, triglyceride glucose-waist-to-height ratio.

The analysis showed that the primary findings were consistent across various subgroups defined by age, race, education, marital status, smoking, CHF, CHD, hypertension, and diabetes, with all *P*-values for interaction greater than 0.05. Besides, when examining the gender groups, it was found that the TyGWHtR and NAFLD had a stronger association in the male subgroup (P-value for interaction <0.05; Figure 4).

## Discussion

In this study, we identified a significant positive nonlinear association between WHtR, TyG, TyGWHtR, WWI, and CMI with the risk of NAFLD in individuals aged 60 and over in the US. Among these indicators, TyGWHtR demonstrated the best predictive capability, with the risk of NAFLD increasing by 28% for every 1-unit increase. Subgroup analysis revealed that the association between TyGWHtR and NAFLD remained robust and was even more pronounced in men. These findings support the identification of TyGWHtR as a simple, single indicator for efficient NAFLD risk stratification in this growing demographic, addressing a key need in public health screening.

As the obesity rate continues to rise, the prevalence of NAFLD is also increasing. Early identification and intervention of high-risk individuals for NAFLD are essential. As a result, numerous biological indicators are being proposed for public health screenings in communities. Traditional indicators of obesity, such as weight, WC, and BMI, are inadequate for differentiating between fat and lean body mass. Consequently, they cannot accurately predict obesity-related diseases, a phenomenon referred to as the "obesity paradox." (43). Our research also indicated that the association between weight, WC, BMI, and NAFLD was not significant, underscoring the widespread limitations of traditional obesity indicators in biological indicator practice.

New obesity-related indicators, such as WWI, CMI, VAI, LAP, TyG, and its derivatives, have significant advantages in predicting NAFLD. These indicators combine body measurements and blood biochemical data, providing a more comprehensive reflection of an individual’s metabolic state. However, the effectiveness of different biomarkers in predicting the disease can vary among other regions and races due to the vast global population and diversity in distribution (44). For example, in the Italian population, the VAI is recognized as the most effective predictor of NAFLD (45). A study by Sheng et al. found that the TyGBMI has the strongest association with NAFLD risk among Japanese adults (36). In Korea, there is a significant correlation between the TyGWC and NAFLD (46). In the United States, adults over the age of 20 show positive associations between WWI and CMI with NAFLD (47, 48). Additionally, research focused on older Chinese individuals has demonstrated a close link between the LAP and the risk of developing NAFLD (49). However, our findings reveal a very weak association between LAP and adults over 60 in the United States, with an OR of about 1. Various factors—such as cultural, dietary, exercise, and genetic differences among populations—contribute to variations in fat distribution, which in turn affects the selection of the most relevant indicators for NAFLD (50, 51). This further emphasizes the importance of choosing different indicators for various demographic groups.

NAFLD includes various conditions characterized by the accumulation of fat within liver cells due to excessive TG synthesis. The onset of NAFLD is linked to several factors, including lipid accumulation, oxidative stress, endoplasmic reticulum stress, and lipid toxicity (52, 53). In a state of IR, the body's ability to prevent lipolysis is diminished. This results in the breakdown of white adipose tissue and a significant release of free fatty acids (FFA). As a consequence, excess FFA is stored in the liver as TG, leading to ectopic lipid deposits and, ultimately, the development of NAFLD (54, 55). The TyG index measures both blood glucose and TG levels, providing a more accurate assessment of IR *in vivo* and serving as an effective predictor for the risk of NAFLD. Additionally, the TyGWHtR incorporates WC and height, offering a more comprehensive understanding of systemic lipid metabolism. TyGWHtR has demonstrated strong predictive effects in various conditions and diseases, including cardiovascular disease, kidney disease, diabetes, and immune system disorders (56–59). Additionally, TyGWHtR is also linked to cardiovascular mortality risk in patients with NAFLD (60). Our study indicated that five indicators (WHtR, TyG, TyGWHtR, WWI, and CMI) show a significant positive association with individuals over 60 years old in the United States, and TyGWHtR demonstrates the most potent predictive effect (AUC: 0.7641, 95%CI: 0.7414-0.7869, Sensitivity: 0.806, Specificity: 0.589). After cross-validation, the association between TyGWHtR and NAFLD remained robust. These results reflect the powerful performance of TyGWHtR in biological indicator application and are therefore increasingly favored by scholars.

Molecular markers associated with obesity and metabolic dysfunction, such as adiponectin, leptin, tumor necrosis factor-alpha (TNF-*α*), and interleukin-6, have been identified as key factors in the development of NAFLD (61–63). Clinical studies have demonstrated a strong correlation between the TyG index and serum adiponectin levels (64). Similarly, significant associations exist between plasma leptin and TyG (65). Research by Leite et al. has shown that the TyG index, along with TNF and interleukin levels, is higher in diabetic patients compared to non-diabetic individuals (66). Therefore, TyG and NAFLD can be associated through metabolite pathway-related molecular markers. Histological evidence presented by Liu et al. through abdominal CT and MRI examinations has confirmed that the degree of hepatic fat accumulation is closely related to the TyG index (67). Therefore, using the TyG index for population health screening and identifying individuals at high risk for NAFLD is scientifically supported from both molecular and histological viewpoints.

Interestingly, given the significant role inflammation plays in the development and progression of NAFLD, we initially expected that SIRI and SII, which represent systemic inflammatory markers, would have a strong predictive power for NAFLD. However, our findings revealed no statistically significant association between SIRI and SII and NAFLD, whether in univariate or multivariate models (*p* for OR> 0.05). We speculate that this may be because SIRI and SII reflect the overall inflammatory state of the body, influenced by various factors such as autoimmune diseases, infections, injuries, and tumors (68–70). As a result, SIRI and SII do not exhibit high specificity in predicting NAFLD. Similarly, while inflammation is a crucial factor in NAFLD, it is not the only one, which helps to explain why SIRI and SII cannot predict the risk of NAFLD.

In our study, we conducted sensitivity analyses to confirm the stability of our conclusions. We first considered potential confounding factors that could affect the results, and thus established three models with adjusted covariates. The results showed that the association between 16 indicators and NAFLD remained stable. Additionally, we performed subgroup analysis and interaction analysis, the results showed that the association between TyGWHtR and NAFLD was very robust (all *p* <0.05). Notably, we found that TyGWHtR had a greater predictive effect in the male population (*p* for interaction < 0.05). This can be attributed to several factors. Firstly, although the incidence of NAFLD in women increases after menopause (71), men typically experience more severe steatosis and steatohepatitis, along with elevated levels of pro-inflammatory and pro-fibrotic cytokines (72). Additionally, men have a higher prevalence of metabolic syndrome, leading to elevated blood glucose and TG levels compared to women (73, 74). Moreover, as men age, changes in WC are more significant than in women, making this a crucial factor (75). Lastly, hormonal changes in women significantly affect fat distribution, especially during reproductive years, while men generally experience stable hormone levels, leading to a greater tendency for visceral fat accumulation at any age (76, 77). These factors collectively explain why the TyGWHtR is more considerable in men.

Compared to other obesity, inflammation, and metabolic indicators, the TyGWHtR is significantly more effective at predicting the risk of NAFLD in older adults aged 60 years and above in the United States. This information can assist public health departments in developing more targeted intervention strategies, such as integrating TyGWHtR into routine senior health check-ups at community centers, or using it to stratify risk and allocate resources for targeted ultrasound screening programs. The advantage of TyGWHtR is its ease of acquisition, as it only requires the calculation of four routine, easily obtainable, and cost-effective indicators: fasting blood glucose, triglycerides, height, and weight. Therefore, it serves as a highly effective risk stratification tool in primary healthcare, particularly for the initial screening of large populations and in resource-limited settings. TyGWHtR can help doctors more accurately identify individuals who require further investigation. For those with a high TyGWHtR (Cut-off Value ≥ 5.276), there should be heightened vigilance for the possibility of NAFLD. If necessary, more detailed liver imaging or even liver biopsy may be required. Its convenience, low cost, and broad applicability make TyGWHtR a promising tool. We propose that its implementation in public health strategies could foster healthy aging by enabling the early detection and prevention of metabolic diseases at a population level.

### Strengths, limitations and future perspectives

#### Strengths

This study comprehensively evaluated 16 indicators from metabolic, immune, inflammatory, and obesity domains to predict NAFLD risk in U.S. adults aged ≥60 years, providing an objective reference for public health screening. It addresses a notable gap in research focused on this age group. The use of high-quality, nationally representative NHANES data and rigorous sensitivity analyses enhances the reliability of our findings regarding TyGWHtR.

### Limitations and future perspectives

This study has some limitations that define its scope and suggest valuable avenues for future research. First, in line with our goal of identifying a simple tool for initial screening, we purposely compared the independent predictive value of multiple indices instead of creating a complex model that involves competitive variable selection. This practical approach, while effective, does not account for potential interactions between variables, representing a trade-off that prioritizes feasibility. Future research should investigate the integration of TyGWHtR into more comprehensive models by incorporating genetic factors, lifestyle, comorbidities, or other novel biomarkers to enhance detection accuracy, thereby addressing more complex public health challenges in the older population. Second, the cross-sectional design precludes causal inference, and NAFLD was defined by FibroScan rather than histology. This necessitates longitudinal studies to validate TyGWHtR's utility in predicting hard liver outcomes and to explore the biological pathways linking it to NAFLD progression. Lastly, our findings in U.S. older adults require validation in other populations. Future research should prioritize external validation in diverse populations and implementation science studies to assess the cost-effectiveness and health outcomes of TyGWHtR-based screening in diverse community health programs.

## Conclusion

The TyGWHtR index is a simple, single indicator that is significantly and independently associated with NAFLD and outperforms other obesity, metabolic, and inflammatory indicators in predicting NAFLD risk among older U.S. adults. Its reliance on routine clinical parameters makes it a highly feasible and efficient tool for large-scale, initial NAFLD screening in public health and primary care practice. Implementing TyGWHtR can facilitate early intervention in high-risk populations, thereby contributing to the goal of healthy aging.

## Data Availability

The datasets presented in this study can be found in online repositories. The names of the repository/repositories and accession number(s) can be found at: https://wwwn.cdc.gov/nchs/nhanes.

## Footnotes

1
https://wwwn.cdc.gov/nchs/nhanes/Default.aspx
