# Supplementary material for: The association between triglyceride glucose-waist-to-height ratio and non-alcoholic fatty liver disease in adults aged over 60 in the United States: a cross-sectional study
Source: Front Public Health. 2026 Jan 21;13:1569324. doi: 10.3389/fpubh.2025.1569324 (PMC12867799; doi:10.3389/fpubh.2025.1569324)
Supplement: Supplementary file 1 [file Data_Sheet_1.PDF]

Table S1. Cross-validation results of the five risk indicators

	AUC	Sensitivity	Specificity
TyGWHtR	0.7816	0.821	0.581
WHtR	0.7639	0.798	0.575
TyG	0.702	0.794	0.456
WWI	0.7082	0.770	0.500
CMI	0.7104	0.819	0.462

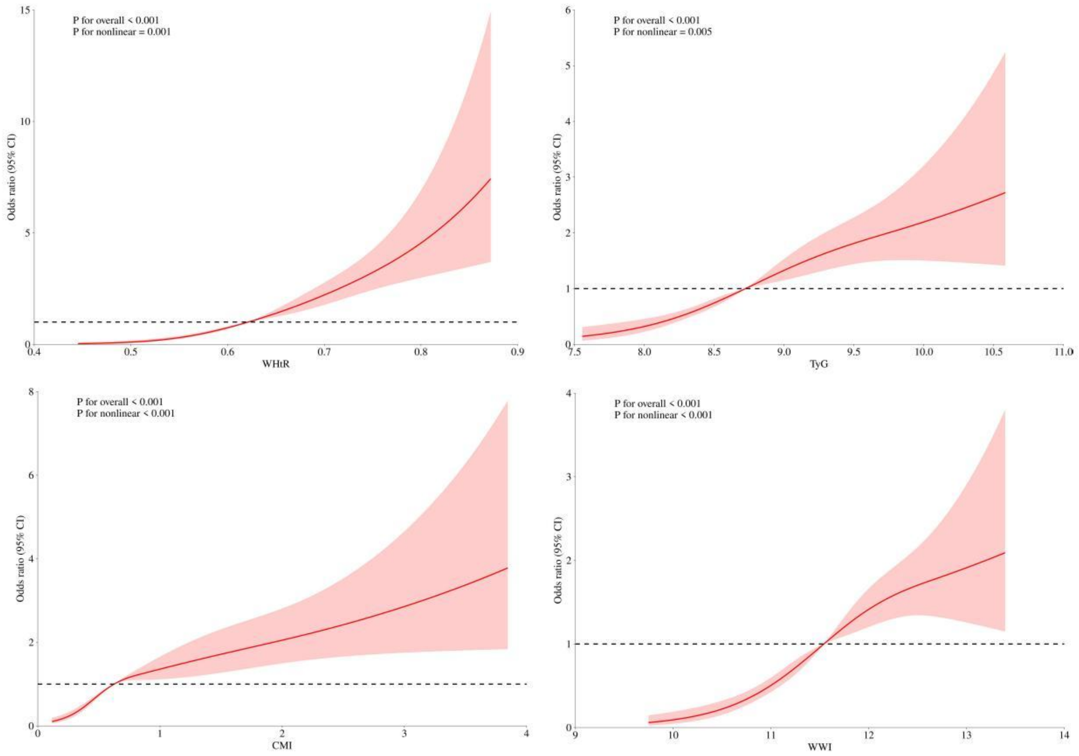

Figure S1. Dose-response relationship between indicators (WHtR, TyG, WWI, CMI) and the risk of NAFLD. The curve plotted by adjusting for covariates including gender, age, race, education, marital status, PIR, smoking, CHF, CHD, hypertension, diabetes, AST and creatinine. The solid line represents the OR value, and the shadow represents 95% CI.
